# Supplementary material for: Risk of Lactic Acidosis in Hospitalized Diabetic Patients Prescribed Biguanides in Japan: A Retrospective Total-Population Cohort Study
Source: Int J Environ Res Public Health. 2023 Mar 29;20(7):5300. doi: 10.3390/ijerph20075300 (PMC10093879; doi:10.3390/ijerph20075300)
Supplement: Supplementary file 1 [file ijerph-20-05300-s001.zip › Supplementary_Table_S1.pdf]

Supplementary Table S1 Pharmaceutical Codes of antidiabetic medications

Types of antidiabetic medication	Pharmaceutical codes
Sulfonylureas	610412056, 610443002, 610443003, 613960002, 613960003, 613960008, 613960017, 613960026, 613960027, 613960028, 613960038, 613960039, 613960078, 620000048, 620002031, 620002032, 620003159, 620003160, 620003947, 620003948, 620006030, 620006890, 620009209, 620871601, 620872002, 620872003, 620872004, 620872009, 620872016, 620873202, 620873301, 620873402, 620873702, 621982701, 621997001, 621997101, 621998701, 621998801, 621998901, 621999001, 621999301, 621999401, 621999701, 621999801, 622000601, 622000701, 622001701, 622001801, 622004701, 622004801, 622005501, 622005601, 622005802, 622009802, 622009901, 622010001, 622011401, 622011501, 622011601, 622011701, 622013401, 622013501, 622013601, 622016001, 622016101, 622017301, 622017401, 622017501, 622017901, 622018001, 622018802, 622020903, 622021003, 622021801, 622021901, 622022001, 622022101, 622023501, 622023601, 622025201, 622025301, 622025801, 622025901, 622026501, 622026601, 622029901, 622030001, 622031401, 622031501, 622033001, 622033101, 622033201, 622033701, 622033801, 622035701, 622035801, 622036002, 622037901, 622038001, 622039901, 622048401, 622048501, 622058801, 622058901, 622059002, 622059102, 622075601, 622088301, 622088401, 622103201, 622114701, 622114801, 622118501, 622122201, 622122301, 622127301, 622127401, 622127501, 622128101, 622137701, 622141101, 622141302, 622143402, 622144001, 622159301, 622169102, 622169301, 622176301, 622177501, 622186201, 622187301, 622190001, 622190801,

	622193301, 622194901, 622198001, 622202201, 622202801, 622205101, 622205501, 622208901, 622211501, 622217701, 622219701, 622221001, 622222001, 622242001, 622246801, 622252501, 622254701, 622271101, 622271201, 622271301, 622313200, 622313300, 622338501, 622338601, 622338701
Meglitinides	622462501, 622462401, 620001908, 620001907, 622053601, 622040901, 622041001, 610432026, 610432027, 622196601, 622119301, 622230001, 622196701, 622119401, 622230101, 610432032, 610432033, 622518201, 622525401, 622515301, 622523401, 622521001, 622518101, 622525301, 622515201, 622523301, 622520901
α-glucosidase inhibitors	610406390, 620002841, 620002843, 620004045, 620004072, 620004071, 620008727, 620008726, 621665301, 621683401, 621673501, 621691201, 622090001, 621689303, 621689001, 621690402, 621690901, 621690203, 620002120, 620004069, 620005557, 620005558, 620005559, 620005560, 620005561, 620008071, 620008072, 620008073, 621953301, 621943301, 620009287, 621896502, 622008602, 620009286, 621896402, 622008502, 620009296, 620009294, 620009293, 622302301, 620009297, 621958801, 620005360, 621785002, 621942202, 620009295, 620009291, 620009289, 620009288, 622302201, 620009292, 621958701, 620005359, 621784902, 621942102, 620009290, 621937201, 621937101, 613960082, 613960081, 622053601, 622432501, 622426601, 622426701, 620003127, 620003128, 620003129, 620002121, 610406391, 621953401, 620004046, 620004070, 620005562, 620008074, 620005563, 620005564, 620005565,

	620008075, 620005566, 621943401, 620008076, 620004073, 620002845, 621690303, 620008729, 620008728, 620004074, 621689403, 621689101, 621691001, 621691601, 621665401, 621683501, 620002847, 622090101, 621690502, 621673601
Biguanides	622517101, 622450401, 622450301, 620004480, 620004502, 620005979, 610463145, 621986401, 621986301, 610444147, 621974701, 622242501, 622070801, 621676001,
	620005570, 622427201, 622421901, 622424401, 622421101, 622412701, 622438401, 622417101, 622432601, 622436301, 622427301, 622422001, 622424501, 622421201, 622448601, 622438501, 622417201, 622432701, 622466601
Thiazolidinediones	621990901, 621991001, 610432040, 610432041, 622048501, 622048401, 622065301, 622061601, 622041402, 622155901, 622063201, 622156901, 622144601, 622167201, 622045401, 622159401, 622056001, 622147501, 622175601, 622071901, 622065401, 622061701, 622041502, 622156001, 622063301, 622157001, 622144701, 622167301, 622045501, 622159501, 622056101, 622147601, 622175701, 622072001, 622320800, 622320900, 622065101, 622042901, 622061401, 622166801, 622182401, 622041202, 622079101, 622155701, 622063001, 622066201, 622164301, 622062302, 622047701, 622049901, 622046801, 622163301, 622053101, 622081801, 622059201, 622045201, 622053801, 622055801, 622147301, 622078301, 622175401, 622071701, 622065201, 622043001, 622061501, 622166901, 622182501, 622041302, 622079201, 622155801, 622063101, 622066301, 622164401, 622062402, 622047801, 622050001, 622046901,

	622163401, 622053201, 622081901, 622059301, 622045301, 622061001, 622055901, 622147401, 622078401, 622175501, 622071801, 621986401, 621986301, 622086101, 622086001
Dipeptidyl peptidase-4 inhibitors	621950901, 621951001, 621951101, 621970601, 621970701, 621970801, 621980701, 621986001, 621986101, 621986201, 622086001, 622086101, 622093501, 622182601, 622201701, 622245601, 622245701, 622277501, 622288401, 622415401, 622415501, 622448901, 622449001, 622450301, 622450401, 622517101
Sodium glucose cotransporter 2 inhibitor	622340101, 622360601, 622401201, 622401301, 622306601, 622306701, 622336801, 622342001, 622341901, 622335701, 622335801
Rapid-acting insulin	621911101, 621911301, 621911201, 620008895, 621926901, 622252701, 620008893, 620008894, 620008916, 640451027, 620007460
Short-acting insulin	620008897, 620000265, 620008909, 620008907, 622114401
Long-acting insulin	622440701, 620008945, 620008943, 620007536, 622198901, 622199001, 622410901, 622484801, 622411001, 621927001, 620008952, 620008953
Immediate-acting insulin	620000266, 620008912, 620008910, 622114501, 620002441, 620007459
Premixed insulin	620002439, 620007461, 620002440, 620007462, 620008915, 620008913, 622114601, 620000269, 620000448, 620008896, 621973201, 621973301, 640453023
Combination-acting insulin	622451001, 622450901
Glucagon-like peptide-1 receptor agonist	622038401, 622038301, 621974801, 622229001, 622406001, 622267001, 622442201
