# Supplementary material for: Risk of Lactic Acidosis in Hospitalized Diabetic Patients Prescribed Biguanides in Japan: A Retrospective Total-Population Cohort Study
Source: Int J Environ Res Public Health. 2023 Mar 29;20(7):5300. doi: 10.3390/ijerph20075300 (PMC10093879; doi:10.3390/ijerph20075300)
Supplement: Supplementary file 1 [file ijerph-20-05300-s001.zip › Supplementary_Table_S2.pdf]

Supplementary Table S2 The diagnosis codes of diabetes

diagnosis in Japanese	diagnosis in English	ICD-10 code	diagnosis code
１型糖尿病	Type 1 diabetes mellitus	E10	2500014
不安定型糖尿病	Brittle diabetes	E10	2500027
緩徐進行１型糖尿病	SPIDDM*	E10	8844022
１型糖尿病性昏睡	Type 1 diabetic coma	E100	8830030
１型糖尿病・昏睡合併あり	Type 1 diabetes mellitus with coma	E100	8841679
緩徐進行１型糖尿病・昏睡合併あり	SPIDDM with coma	E100	8844026
１型糖尿病性低血糖性昏睡	Hypoglycemia in the context of type 1 diabetes mellitus	E100	8845065
１型糖尿病性ケトアシドーシス	Diabetic ketoacidosis in type 1 diabetes mellitus	E101	8830028
１型糖尿病・ケトアシドーシス合併あり	Type 1 diabetes mellitus with ketoacidosis	E101	8841680
緩徐進行１型糖尿病・ケトアシドーシス合併あり	SPIDDM with ketoacidosis	E101	8844025

劇症１型糖尿病	Fulminant type 1 diabetes mellitus	E101	8844045
１型糖尿病性アシドーシス	Diabetic ketoacidosis in type 1 diabetes mellitus	E101	8845044
１型糖尿病性アセトン血症	Type 1 diabetic acetone hyperlipoproteinemia	E101	8845045
１型糖尿病性腎症	Type 1 diabetic nephropathy	E102	8830031
１型糖尿病・腎合併症あり	Type 1 diabetes mellitus with diabetic nephropathy	E102	8841681
１型糖尿病性腎症第１期	Type 1 diabetic nephropathy phase 1	E102	8843983
１型糖尿病性腎症第２期	Type 1 diabetic nephropathy phase 2	E102	8843984
１型糖尿病性腎症第３期	Type 1 diabetic nephropathy phase 3	E102	8843985
１型糖尿病性腎症第３期Ａ	Type 1 diabetic nephropathy phase 3A	E102	8843986
１型糖尿病性腎症第３期Ｂ	Type 1 diabetic nephropathy phase 3B	E102	8843987
１型糖尿病性腎症第４期	Type 1 diabetic nephropathy phase 4	E102	8843988

１型糖尿病性腎症第５期	Type 1 diabetic nephropathy phase 5	E102	8843989
緩徐進行１型糖尿病・腎合併症あり	SPIDDM with nephropathy	E102	8844028
１型糖尿病性腎硬化症	Type 1 diabetic nephrosclerosis	E102	8845058
１型糖尿病性腎不全	Type 1 diabetic kidney failure	E102	8845059
１型糖尿病性網膜症	Type 1 diabetic retinopathy	E103	8830033
１型糖尿病・眼合併症あり	Type 1 diabetes mellitus with eye complication	E103	8841682
１型糖尿病性黄斑浮腫	Type 1 diabetic macular edema	E103	8843982
緩徐進行１型糖尿病・眼合併症あり	SPIDDM with eye complication	E103	8844024
１型糖尿病性白内障	Type 1 diabetic cataracts	E103	8844346
増殖性糖尿病性網膜症・１型糖尿病	Proliferative diabetic retinopathy, type 1 diabetes	E103	8844536
１型糖尿病黄斑症	Type 1 diabetic macular disease	E103	8845043

１型糖尿病性眼筋麻痺	Type 1 diabetic eye muscle paralysis	E103	8845049
１型糖尿病性虹彩炎	Type 1 diabetic iritis	E103	8845053
１型糖尿病性中心性網膜症	Type 1 diabetic central retinopathy	E103	8845064
１型糖尿病性ニューロパチー	Type 1 diabetic neuropathy	E104	8830032
１型糖尿病・神経学的合併症あり	Type 1 diabetes mellitus with neurological complications	E104	8841683
緩徐進行１型糖尿病・神経学的合併症あり	SPIDDM with neurological complications	E104	8844027
１型糖尿病性筋萎縮症	Type 1 diabetic muscular atrophy	E104	8845050
１型糖尿病性神経因性膀胱	Type 1 diabetic neuropathic bladder	E104	8845055
１型糖尿病性神経痛	Type 1 diabetic neuralgia	E104	8845056
１型糖尿病性自律神経ニューロパチー	Type 1 diabetic autonomic neuropathy	E104	8845057
１型糖尿病性多発ニューロパチー	Type 1 diabetic polyneuropathy	E104	8845062

１型糖尿病性単ニューロパチー	Type 1 diabetic mononeuropathy	E104	8845063
１型糖尿病性末梢神経障害	Type 1 diabetic peripheral neuropathy	E104	8845071
１型糖尿病・末梢循環合併症あり	Type 1 diabetes mellitus with peripheral circulation complications	E105	8841684
１型糖尿病性壊疽	Type 1 diabetic gangrene	E105	8843105
緩徐進行１型糖尿病・末梢循環合併症あり	SPIDDM with peripheral circulation complications	E105	8844031
１型糖尿病性潰瘍	Type 1 diabetic ulcer	E105	8845046
１型糖尿病性血管障害	Type 1 diabetic vascular disease	E105	8845051
１型糖尿病性動脈硬化症	Type 1 diabetic atherosclerosis	E105	8845066
１型糖尿病性動脈閉塞症	Type 1 diabetic arterial occlusion	E105	8845067
１型糖尿病性末梢血管症	Type 1 diabetic peripheral vascular disease	E105	8845069
１型糖尿病性末梢血管障害	Type 1 diabetic peripheral vascular disease	E105	8845070

１型糖尿病・関節合併症あり	Type 1 diabetes mellitus with joint complications	E106	8841685
１型糖尿病・糖尿病性合併症あり	Type 1 diabetes mellitus with diabetic complications	E106	8841686
緩徐進行１型糖尿病・関節合併症あり	SPIDDM with joint complications	E106	8844023
１型糖尿病性水疱	Type 1 diabetic blister	E106	8844626
１型糖尿病性浮腫性硬化症	Type 1 diabetic edematous sclerosis	E106	8844627
１型糖尿病性肝障害	Type 1 diabetic liver injury	E106	8845047
１型糖尿病性関節症	Type 1 diabetic arthropathy	E106	8845048
１型糖尿病性高コレステロール血症	Type 1 diabetic hypercholesterolemia	E106	8845052
１型糖尿病性骨症	Type 1 diabetic osteopathy	E106	8845054
１型糖尿病性精神障害	Type 1 diabetic mental disorder	E106	8845060
１型糖尿病性そう痒症	Type 1 diabetic pruritus	E106	8845061

１型糖尿病性皮膚障害	Type 1 diabetic skin disorder	E106	8845068
１型糖尿病性胃腸症	Type 1 diabetic gastroenteritis	E106	8845842
１型糖尿病・多発糖尿病性合併症あり	Type 1 diabetes mellitus with multiple diabetic complications	E107	8841687
緩徐進行１型糖尿病・多発糖尿病性合併症あり	SPIDDM with multiple diabetic complications	E107	8844029
１型糖尿病・糖尿病性合併症なし	Type 1 diabetes mellitus without diabetic complications	E109	8841688
緩徐進行１型糖尿病・糖尿病性合併症なし	SPIDDM without diabetic complications	E109	8844030
インスリン抵抗性糖尿病	Insulin resistant diabetes mellitus	E11	2500001
２型糖尿病	Type 2 diabetes mellitus	E11	2500015
安定型糖尿病	Stable diabetes mellitus	E11	8830405
若年２型糖尿病	Juvenile type 2 diabetes	E11	8835244
２型糖尿病性昏睡	Type 2 diabetic coma	E110	8830041

２型糖尿病・昏睡合併あり	Type 2 diabetes mellitus with coma	E110	8841689
２型糖尿病性低血糖性昏睡	Hypoglycemic coma in the context of type 2 diabetes mellitus	E110	8845094
２型糖尿病性ケトアシドーシス	Type 2 diabetic ketoacidosis	E111	8830040
２型糖尿病・ケトアシドーシス合併あり	Type 2 diabetes mellitus with ketoacidosis	E111	8841690
２型糖尿病性アシドーシス	Type 2 diabetic acidosis	E111	8845073
２型糖尿病性アセトン血症	Type 2 diabetic acetone hyperlipoproteinemia	E111	8845074
２型糖尿病性腎症	Type 2 diabetic nephropathy	E112	8830042
２型糖尿病・腎合併症あり	Type 2 diabetes mellitus with diabetic nephropathy	E112	8841691
２型糖尿病性腎症第１期	Type 2 diabetic nephropathy phase 1	E112	8843991
２型糖尿病性腎症第２期	Type 2 diabetic nephropathy phase 2	E112	8843992
２型糖尿病性腎症第３期	Type 2 diabetic nephropathy phase 3	E112	8843993

２型糖尿病性腎症第３期Ａ	Type 2 diabetic nephropathy phase 3A	E112	8843994
２型糖尿病性腎症第３期Ｂ	Type 2 diabetic nephropathy phase 3B	E112	8843995
２型糖尿病性腎症第４期	Type 2 diabetic nephropathy phase 4	E112	8843996
２型糖尿病性腎症第５期	Type 2 diabetic nephropathy phase 5	E112	8843997
２型糖尿病性腎硬化症	Type 2 diabetic nephrosclerosis	E112	8845087
２型糖尿病性腎不全	Type 2 diabetic kidney failure	E112	8845088
２型糖尿病性網膜症	Type 2 diabetic retinopathy	E113	8830045
２型糖尿病・眼合併症あり	Type 2 diabetes mellitus with eye complications	E113	8841692
２型糖尿病性黄斑浮腫	Type 2 diabetic macular edema	E113	8843990
２型糖尿病性白内障	Type 2 diabetic cataracts	E113	8844347
増殖性糖尿病性網膜症・２型糖尿病	Proliferative diabetic retinopathy, type 2 diabetes	E113	8844537

2 型糖尿病黄斑症	Type 2 diabetic macular disease	E113	8845072
2 型糖尿病性眼筋麻痺	Type 2 diabetic eye muscle paralysis	E113	8845078
2 型糖尿病性虹彩炎	Type 2 diabetic iritis	E113	8845082
2 型糖尿病性中心性網膜症	Type 2 diabetic central retinopathy	E113	8845093
2 型糖尿病性ニューロパチー	Type 2 diabetic neuropathy	E114	8830043
2 型糖尿病性ミオパチー	Type 2 diabetic myopathy	E114	8830044
2 型糖尿病・神経学的合併症あり	Type 2 diabetes mellitus with neurological complications	E114	8841693
2 型糖尿病性筋萎縮症	Type 2 diabetic muscular atrophy	E114	8845079
2 型糖尿病性神経因性膀胱	Type 2 diabetic neuropathic bladder	E114	8845084
2 型糖尿病性神経痛	Type 2 diabetic neuralgia	E114	8845085
2 型糖尿病性自律神経ニューロパチー	Type 2 diabetic autonomic neuropathy	E114	8845086

2型糖尿病性多発ニューロパチー	Type 2 diabetic polyneuropathy	E114	8845091
2型糖尿病性単ニューロパチー	Type 2 diabetic mononeuropathy	E114	8845092
2型糖尿病性末梢神経障害	Type 2 diabetic peripheral neuropathy	E114	8845100
2型糖尿病・末梢循環合併症あり	Type 2 diabetes mellitus with peripheral circulation complications	E115	8841694
2型糖尿病性壊疽	Type 2 diabetic gangrene	E115	8843106
2型糖尿病性潰瘍	Type 2 diabetic ulcer	E115	8845075
2型糖尿病性血管障害	Type 2 diabetic vascular disease	E115	8845080
2型糖尿病性動脈硬化症	Type 2 diabetic atherosclerosis	E115	8845095
2型糖尿病性動脈閉塞症	Type 2 diabetic arterial occlusion	E115	8845096
2型糖尿病性末梢血管症	Type 2 diabetic peripheral vascular disease	E115	8845098
2型糖尿病性末梢血管障害	Type 2 diabetic peripheral vascular disease	E115	8845099

２型糖尿病・関節合併症あり	Type 2 diabetes mellitus with joint complications	E116	8841695
２型糖尿病・糖尿病性合併症あり	Type 2 diabetes mellitus with diabetic complications	E116	8841696
２型糖尿病性水疱	Type 2 diabetic blister	E116	8844628
２型糖尿病性浮腫性硬化症	Type 2 diabetic edematous sclerosis	E116	8844629
２型糖尿病性肝障害	Type 2 diabetic liver injury	E116	8845076
２型糖尿病性関節症	Type 2 diabetic arthropathy	E116	8845077
２型糖尿病性高コレステロール血症	Type 2 diabetic hypercholesterolemia	E116	8845081
２型糖尿病性骨症	Type 2 diabetic osteopathy	E116	8845083
２型糖尿病性精神障害	Type 2 diabetic mental disorder	E116	8845089
２型糖尿病性そう痒症	Type 2 diabetic pruritus	E116	8845090
２型糖尿病性皮膚障害	Type 2 diabetic skin disorder	E116	8845097

２型糖尿病性胃腸症	Type 2 diabetic gastroenteritis	E116	8848108
２型糖尿病・多発糖尿病性合併症あり	Type 2 diabetes mellitus with multiple diabetic complications	E117	8841697
２型糖尿病・糖尿病性合併症なし	Type 2 diabetes mellitus without diabetic complications	E119	8841698
栄養不良関連糖尿病	Malnutrition-related diabetes mellitus	E12	2500037
膵性糖尿病	Pancreatic diabetes mellitus	E13	2500024
ステロイド糖尿病	Steroid diabetes mellitus	E13	2509003
二次性糖尿病	Secondary diabetes mellitus	E13	2509004
ウイルス性糖尿病	Viral diabetes mellitus	E13	8830756
薬剤性糖尿病	Drug-induced diabetes mellitus	E13	8840710
ウイルス性糖尿病・昏睡合併あり	Viral diabetes mellitus with coma	E130	8843122
膵性糖尿病・昏睡合併あり	Pancreatic diabetes mellitus with coma	E130	8843377

ステロイド糖尿病・昏睡合併あり	Steroid diabetes mellitus with coma	E130	8843390
二次性糖尿病・昏睡合併あり	Secondary diabetes mellitus with coma	E130	8843450
薬剤性糖尿病・昏睡合併あり	Drug-induced diabetes mellitus with coma	E130	8843621
ウイルス性糖尿病・ケトアシドーシス合併あり	Viral diabetes mellitus with ketoacidosis	E131	8843121
膵性糖尿病・ケトアシドーシス合併あり	Pancreatic diabetes mellitus with ketoacidosis	E131	8843376
ステロイド糖尿病・ケトアシドーシス合併あり	Steroid diabetes mellitus with ketoacidosis	E131	8843389
二次性糖尿病・ケトアシドーシス合併あり	Secondary diabetes mellitus with ketoacidosis	E131	8843449
薬剤性糖尿病・ケトアシドーシス合併あり	Drug-induced diabetes mellitus with ketoacidosis	E131	8843620
ウイルス性糖尿病・腎合併症あり	Viral diabetes mellitus with renal complications	E132	8843124
膵性糖尿病・腎合併症あり	Pancreatic diabetes mellitus with renal complications	E132	8843379
ステロイド糖尿病・腎合併症あり	Steroid diabetes mellitus with renal complications	E132	8843392

二次性糖尿病・腎合併症あり	Secondary diabetes mellitus with renal complications	E132	8843452
薬剤性糖尿病・腎合併症あり	Drug-induced diabetes mellitus with renal complications	E132	8843623
ウイルス性糖尿病・眼合併症あり	Viral diabetes mellitus with eye complications	E133	8843120
膵性糖尿病・眼合併症あり	Pancreatic diabetes mellitus with eye complications	E133	8843375
ステロイド糖尿病・眼合併症あり	Steroid diabetes mellitus with eye complications	E133	8843388
二次性糖尿病・眼合併症あり	Secondary diabetes mellitus with eye complications	E133	8843448
薬剤性糖尿病・眼合併症あり	Drug-induced diabetes mellitus with eye complications	E133	8843619
ウイルス性糖尿病・神経学的合併症あり	Viral diabetes mellitus with neurological complications	E134	8843123
膵性糖尿病・神経学的合併症あり	Pancreatic diabetes mellitus with neurological complications	E134	8843378
ステロイド糖尿病・神経学的合併症あり	Steroid diabetes mellitus with neurological complications	E134	8843391
二次性糖尿病・神経学的合併症あり	Secondary diabetes mellitus with neurological complications	E134	8843451

薬剤性糖尿病・神経学的合併症あり	Drug-induced diabetes mellitus with neurological complications	E134	8843622
ウイルス性糖尿病・末梢循環合併症あり	Viral diabetes mellitus with peripheral circulatory complications	E135	8843128
膵性糖尿病・末梢循環合併症あり	Pancreatic diabetes mellitus with peripheral circulatory complications	E135	8843383
ステロイド糖尿病・末梢循環合併症あり	Steroid diabetes mellitus with peripheral circulatory complications	E135	8843396
二次性糖尿病・末梢循環合併症あり	Secondary diabetes mellitus with peripheral circulatory complications	E135	8843456
薬剤性糖尿病・末梢循環合併症あり	Drug-induced diabetes mellitus with peripheral circulatory complications	E135	8843627
ウイルス性糖尿病・糖尿病性合併症あり	Viral diabetes mellitus with diabetic complications	E136	8843126
膵性糖尿病・糖尿病性合併症あり	Pancreatic diabetes mellitus with diabetic complications	E136	8843381
ステロイド糖尿病・糖尿病性合併症あり	Steroid diabetes mellitus with diabetic complications	E136	8843394
二次性糖尿病・糖尿病性合併症あり	Secondary diabetes mellitus with diabetic complications	E136	8843454
薬剤性糖尿病・糖尿病性合併症あり	Drug-induced diabetes mellitus with diabetic complications	E136	8843625

ウイルス性糖尿病・多発糖尿病性合併症あり	Viral diabetes mellitus with multiple diabetic complications	E137	8843125
膵性糖尿病・多発糖尿病性合併症あり	Pancreatic diabetes mellitus with multiple diabetic complications	E137	8843380
ステロイド糖尿病・多発糖尿病性合併症あり	Steroid diabetes mellitus with multiple diabetic complications	E137	8843393
二次性糖尿病・多発糖尿病性合併症あり	Secondary diabetes mellitus with multiple diabetic complications	E137	8843453
薬剤性糖尿病・多発糖尿病性合併症あり	Drug-induced diabetes mellitus with multiple diabetic complications	E137	8843624
ウイルス性糖尿病・糖尿病性合併症なし	Viral diabetes mellitus without diabetic complications	E139	8843127
膵性糖尿病・糖尿病性合併症なし	Pancreatic diabetes mellitus without diabetic complications	E139	8843382
ステロイド糖尿病・糖尿病性合併症なし	Steroid diabetes mellitus without diabetic complications	E139	8843395
二次性糖尿病・糖尿病性合併症なし	Secondary diabetes mellitus without diabetic complications	E139	8843455
薬剤性糖尿病・糖尿病性合併症なし	Drug-induced diabetes mellitus without diabetic complications	E139	8843626
糖尿病	Diabetes mellitus	E14	2500013

糖尿病合併症	Diabetic complications	E14	2507028
糖尿病性昏睡	Diabetic coma	E140	2502006
糖尿病性低血糖性昏睡	Hypoglycemic coma in the context of diabetes mellitus	E140	8838076
糖尿病性アシドーシス	Diabetic acidosis	E141	2501002
糖尿病性アセトン血症	Diabetic acetonemia	E141	2501003
糖尿病性ケトアシドーシス	Diabetic ketoacidosis	E141	2501005
糖尿病性腎症	Diabetic nephropathy	E142	2503005
糖尿病性腎不全	Diabetic renal failure	E142	2503007
糖尿病性腎硬化症	Diabetic nephrosclerosis	E142	8838071
糖尿病性虹彩炎	Diabetic iritis	E143	2504004
糖尿病性中心性網膜症	Diabetic central retinopathy	E143	2504005

糖尿病性白内障	Diabetic cataract	E143	2504006
増殖性糖尿病性網膜症	Proliferative diabetic retinopathy	E143	2504010
糖尿病黄斑症	Diabetic maculopathy	E143	2504012
糖尿病網膜症	Diabetic retinopathy	E143	2504013
糖尿病性眼筋麻痺	Diabetic ophthalmoplegia	E143	8838065
糖尿病黄斑浮腫	Diabetic macular edema	E143	8844089
糖尿病性神経痛	Diabetic neuralgia	E144	2505011
糖尿病性末梢神経障害	Diabetic peripheral neuropathy	E144	2505018
糖尿病性筋萎縮症	Diabetic muscular atrophy	E144	2505021
糖尿病性神経因性膀胱	Diabetic neuropathic bladder	E144	8838069
糖尿病性自律神経ニューロパチー	Diabetic autonomic neuropathy	E144	8838070

糖尿病性多発ニューロパチー	Diabetic polyneuropathy	E144	8838074
糖尿病性単ニューロパチー	Diabetic mononeuropathy	E144	8838075
糖尿病性ニューロパチー	Diabetic neuropathy	E144	8838078
糖尿病足病変	Diabetic foot lesion	E144	8848634
糖尿病性神経障害性疼痛	Diabetic neuropathic pain	E144	8848768
糖尿病性壊疽	Diabetic gangrene	E145	2506006
糖尿病性動脈閉塞症	Diabetic arterial occlusion	E145	2506011
糖尿病性潰瘍	Diabetic ulcer	E145	8838063
糖尿病性血管障害	Diabetic angiopathy	E145	8838066
糖尿病性動脈硬化症	Diabetic arteriosclerosis	E145	8838077
糖尿病性末梢血管症	Diabetic peripheral vascular disease	E145	8838079

糖尿病性末梢血管障害	Diabetic peripheral vascular disease	E145	8838080
糖尿病足壊疽	Diabetic foot gangrene	E145	8848632
糖尿病足潰瘍	Diabetic foot ulcer	E145	8848633
糖尿病性関節症	Diabetic arthropathy	E146	2507025
糖尿病性皮膚障害	Diabetic skin disorders	E146	2507029
糖尿病性肝障害	Diabetic liver injury	E146	8838064
糖尿病性高コレステロール血症	Diabetic hypercholesterolemia	E146	8838067
糖尿病性骨症	Diabetic osteopathy	E146	8838068
糖尿病性精神障害	Diabetic mental disorder	E146	8838072
糖尿病性そう痒症	Diabetic pruritus	E146	8838073
糖尿病性水疱	Diabetic blister	E146	8844652

糖尿病性浮腫性硬化症	Diabetic edematous sclerosis	E146	8844653
高血糖高浸透圧症候群	Hyperglycemia hyperosmolarity syndrome	E146	8845128
糖尿病・糖尿病性合併症なし	Diabetes mellitus without diabetic complications	E149	8843439
非糖尿病性低血糖性昏睡	Hypoglycemic coma not in the context of diabetes mellitus	E15	8839324
果糖尿症	Levulosuria	E741	8831401
本態性果糖尿症	Essential levulosuria	E741	8840104
良性果糖尿症	Benign levulosuria	E741	8841021
腎性糖尿	Renal glycosuria	E748	2714002
青銅性糖尿病	Bronze diabetes mellitus	E831	8835941
膵全摘後二次性糖尿病	Secondary diabetes after pancreatectomy	E891	8835685
１型糖尿病合併妊娠	Pregnancy with type 1 diabetes	O240	8830029

２型糖尿病合併妊娠	Pregnancy with type 2 diabetes	O241	8830039
妊娠糖尿病	Pregnancy diabetes mellitus	O244	6489003
妊娠中の糖尿病	Overt diabetes in pregnancy	O249	8838621
妊娠中の耐糖能低下	Impaired glucose tolerance in pregnancy	O998	8838619
妊娠糖尿病母体児症候群	Gestational diabetes maternal syndrome	P700	8838633
糖尿病母体児	Diabetes maternal infant	P701	8838081
新生児一過性糖尿病	Neonatal transient diabetes mellitus	P702	7751001
新生児糖尿病	Neonatal diabetes mellitus	P702	7751002
新生児一過性高血糖症	Neonatal transient hyperglycemia	P708	8844233
境界型糖尿病	Borderline type diabetes mellitus	R730	2500031
耐糖能異常	Impaired glucose tolerance	R730	2713009

化学的糖尿病	Chemical diabetes mellitus	R730	8831132
潜在性糖尿病	Latent diabetes mellitus	R730	8836104
前糖尿病	Pre-diabetes mellitus	R730	8836563
高血糖症	Hyperglycemia	R739	8833419
一過性糖尿	Transient diabetes mellitus	R81	7915002
五炭糖尿症	L-Xylulosuria	R81	7915003
高血糖性糖尿	Hypoglycemic glycosuria	R81	8833420
食事性糖尿	Dietary glycosuria	R81	8834843
情動性糖尿	Emotional glycosuria	R81	8835464
正常血糖性糖尿	Euglycemic glycosuria	R81	8835871
糖尿	Glycosuria	R81	8838062

* SPIDDM means “Slowly Progressive Insulin-Dependent Diabetes Mellitus”
